# Comparative Metabolomics Reveals L-Theanine as a Candidate Contributor to Cultivar-Specific Differences in PTZ-Induced Hyperactivity in Zebrafish

**DOI:** 10.3390/foods15213992

**Published:** 2026-11-07

**Authors:** Qiujian Huang, Fengshui Yang, Salome Njeri Ndombi, Huajuan Cheng, Ying Xu, Lili Zeng, Lilian Kerio, Su’e Lin, Xiufeng Yan, Zhengzhen Li, Xin Li

**Affiliations:** 1 College of Life and Environmental Science, Wenzhou University, Wenzhou 325035, China; 25461047007.wzu@vip.163.com (Q.H.);; 2 Key Laboratory of Tea Quality and Safety Control, Ministry of Agriculture and Rural Affairs, Tea Research Institute, Chinese Academy of Agricultural Sciences, Hangzhou 310008, China; fengshuiy@163.com (F.Y.); lixin@tricaas.com (X.L.); 3 College of Food Science, Southwest University, Chongqing 400715, China; 4 Bureau of Agriculture and Rural Affairs in Anji County, Huzhou 313399, China; 5 Kenya Agricultural and Livestock Research Organization Tea Research Institute, Kericho 82020200, Kenya

**Keywords:** L-theanine, caffeine, hyperactivity, metabolomics, GABA, melatonin

## Abstract

Hyperactivity and excessive neuronal excitation may lead to sleep disturbances and affect neurological health and overall quality of life, highlighting the need for safe, natural remedies. L-theanine, a natural bioactive compound in tea, has attracted interest for its potential effects on behavioral activity, although its effects on PTZ-induced locomotor responses in zebrafish remain unclear. Given the substantial variation in L-theanine accumulation among tea cultivars, we compared the metabolomic profiles of *Camellia sinensis* cultivars ‘Baiye 1’ (BY) and ‘Longjing 43’ (LJ43) and found that BY accumulated higher levels of L-theanine than LJ43. BY tea infusion exhibited stronger effects on pentylenetetrazole (PTZ)-induced hyperactivity in zebrafish, while LJ43 showed limited efficacy. Administering exogenous L-theanine, either alone or in combination with GABA or melatonin, alleviated hyperactivity and partially modulated the expression of *gabra1* and *mtnr1aa* in zebrafish. Molecular docking revealed putative structural interactions of L-theanine with both GABRA1 and MTNR1AA, suggesting that GABA- and melatonin-related receptors may be involved in its attenuation of PTZ-induced hyperactivity. These findings highlight L-theanine as a potential contributor to the attenuation of PTZ-induced hyperactivity. Considering the complex composition of tea infusions, the distinct behavioral responses between BY and LJ43 may reflect the combined effects of multiple bioactive constituents, among which L-theanine may be one plausible contributor.

## 1. Introduction

Hyperactivity and overexcitation are influenced by multiple factors, including psychological stress, caffeine consumption, and the balance between excitatory and inhibitory neurotransmission [1,2]. Persistent sleep disruptions severely compromise physical health, mental health and overall quality of life [3]. However, conventional sedative-hypnotic pharmacotherapies are limited by adverse effects and risks associated with long-term use [4]. Therefore, identifying safe, naturally derived compounds that can relieve excessive arousal and hyperactivity has become an important research focus. Tea (*Camellia sinensis*), a globally preeminent non-alcoholic beverage, is highly valued not only for its distinctive flavor but also for its diverse health-promoting functions [5]. The sleep-related effects of tea are mainly attributed to multiple naturally occurring bioactive constituents, particularly L-theanine and caffeine [6,7]. Among these compounds, L-theanine has attracted increasing attention due to its favorable safety profile [8].

Accumulating evidence indicates that tea constituents such as L-theanine and caffeine may exert contrasting effects on neural arousal and behavioral activity [9,10]. As a structural analogue of glutamine, L-theanine can cross the blood–brain barrier to attenuate hyper-neuronal excitation through modulation of glutamatergic neurotransmission [11,12], thereby potentially contributing to neural relaxation and the attenuation of excessive arousal. In contrast, caffeine acts as a well-established central nervous system stimulant by competitively antagonizing central adenosine receptors. This receptor antagonism suppresses adenosine-mediated neuronal inhibition, thereby sustaining cortical arousal and wakefulness [13,14]. Clinical studies have shown that L-theanine supplementation can shorten sleep latency and improve sleep quality [9], whereas caffeine can prolong sleep latency and reduce total sleep duration [10]. Collectively, these findings underscore the contrasting effects of L-theanine and caffeine on alleviation of hyperactivity.

Previous studies have shown that differences in tea characteristics and chemical composition can influence the biological activities of tea infusions [6]. Moreover, the composition and relative abundance of bioactive compounds vary among cultivars, resulting in distinct sleep-modulating effects [15]. ‘Baiye 1’ (BY) and ‘Longjing 43’ (LJ43) are two representative tea cultivars with contrasting chemical characteristics [16]. BY generally accumulates elevated concentrations of L-theanine, yielding a distinct fresh and umami flavor profile, whereas LJ43 exhibits a distinct metabolite profile balance with a higher caffeine-to-theanine ratio [17]. However, the differential effects of BY and LJ43 on hyperactivity remain largely unexplored. Previous studies have focused on the individual effects of single bioactive compounds such as L-theanine or caffeine [18], whereas their potential interactions with endogenous neuroactive molecules, including GABA and melatonin, remain insufficiently explored.

Zebrafish (*Danio rerio*) serve as a well-established model organism in sleep research due to their compatibility with high-throughput phenotypic screening and neuropharmacological evaluation of bioactive compounds [19,20]. In particular, pentylenetetrazole (PTZ) is widely used to induce neuronal hyperexcitability and seizure-like behaviors in zebrafish, often accompanied by increased locomotor activity. Therefore, PTZ-induced zebrafish hyperactivity provides a useful in vivo model for evaluating the effects of tea cultivars and their bioactive constituents on locomotor responses [21]. In this study, we aimed to compare the metabolomic profiles of ‘Baiye 1’ (BY) and ‘Longjing 43’ (LJ43) to identify candidate metabolites potentially associated with their differential behavioral effects. L-theanine was subsequently selected for further investigation based on its differential accumulation between the two cultivars. We then investigated the effects of L-theanine alone and in combination with GABA, melatonin, or caffeine on PTZ-associated hyperactivity, and explored the effects of *gabra1* and *mtnr1aa* gene expression. By integrating comparative compositional profiling of tea constituents with the in vivo functional validation of individual bioactive compounds, this study provides insights into the metabolic factors associated with modulation of PTZ-induced hyperactivity. These findings may facilitate the identification of tea cultivars with potential calming properties and support the development of functional tea-based products.

## 2. Materials and Methods

### 2.1. Plant Material and Pre-Processing

Fresh shoots (one bud and two leaves) were harvested from the tea cultivars ‘Baiye 1’ and ‘Longjing 43’ grown in Anji County, Zhejiang Province, China. The samples were processed into green tea using a uniform, standardized manufacturing procedure involving spreading, high-temperature fixation, rolling, pan-firing, and drying. The resulting green tea samples were designated BY and LJ43, respectively, and stored at 4 °C in a sealed container protected from light prior to subsequent analysis.

### 2.2. Metabolomic Analysis and L-Theanine Quantification

Metabolomic analysis was performed following a previously reported method [22]. Tea samples were ground into powder, and 100 mg of each sample was extracted overnight at 4 °C using 10 mL of methanol (Aladdin, Shanghai, China)/water (3:1, *v*/*v*). Three independent biological replicates were prepared for each tea sample. The extract was centrifuged at 10,000 rpm for 10 min at 4 °C using an Expert 16K-R centrifuge (Xinao, Hunan, China), and the supernatant was filtered through a 0.22 μm microporous membrane prior to instrumental analysis. Quality control (QC) samples were prepared by pooling 80 μL aliquots from each biological sample to monitor analytical reproducibility and instrument stability. Ultra-high-performance liquid chromatography–mass spectrometry (UHPLC-MS) was performed on a Q-Exactive MS (Thermo Fisher Scientific, Waltham, MA, USA) coupled with an UltiMate 3000 UHPLC (Thermo Fisher Scientific, Waltham, MA, USA). Chromatographic separation was achieved using a Waters Acquity UPLC HSS T3 column (2.1 mm × 100 mm, 1.8 μm, Waters, Milford, MA, USA). Mobile phase A consisted of 0.1% aqueous formic acid solution, and mobile phase B was 0.1% formic acid in acetonitrile (Burdick & Jackson, Muskegon, MI, USA). The column temperature was 35 °C, and the flow rate was 300 μL/min. Mass spectra were detected simultaneously in both electrospray ionization (ESI) positive and negative modes. The scanning mode was Full MS/dd-MS2 (TopN), and the scanning range was *m*/*z* 60–900.

Raw data were acquired using Xcalibur v.2.1.1 and processed using Compound Discoverer v.3.2 (Thermo Fisher Scientific, Waltham, MA, USA), including baseline correction, smoothing, noise reduction, peak detection, and alignment. Features were filtered using a mass accuracy tolerance of ≤5 ppm, retention time deviation of ≤0.2 min, peak intensity ≥ 200,000, and signal-to-noise ratio > 3. Metabolomic data were processed using Compound Discoverer v.3.2 (Thermo Fisher Scientific, Waltham, MA, USA). Metabolites were annotated by comparing accurate mass measurements and MS/MS fragmentation patterns with available reference spectral information. Data preprocessing included quantile normalization, cube root transformation, and mean centering. Differential metabolites between BY and LJ43 were identified based on a variable importance in projection (VIP) value > 1 and an unadjusted *p*-value < 0.05. Fold change (FC) and log_2_FC values were calculated to characterize the magnitude and direction of metabolite abundance differences, and false discovery rate (FDR)-adjusted *p*-values were calculated to assess the effects of multiple testing. Metabolite abundances were further normalized using Z-score transformation for heatmap visualization. For L-theanine quantification, tea samples were extracted with water in a separate experiment. L-theanine concentrations were calculated by validating sample peak areas against reference standard calibrations.

### 2.3. Determination of Total Free Amino Acid and Tea Polyphenol Content

Three 0.1-g tea powder samples (biological replicates) were each extracted with 5 mL of boiling distilled water in a water bath at 100 °C for 45 min. The mixtures were then centrifuged at 12,000 rpm for 10 min at 4 °C, and the supernatants were collected for subsequent analysis. Total free amino acid content was determined using the ninhydrin colorimetric method based on GB/T 8314-2013. Then, 0.1 mL of the supernatant was mixed with 50 μL of 2% ninhydrin solution and 50 μL of pH 8.0 phosphate buffer in a 10-mL centrifuge tube in triplicate. The mixture was heated in a boiling water bath for 5 min, rapidly cooled in ice water, and diluted with 4.8 mL of distilled water. After thorough mixing, the absorbance was measured at 570 nm. The assay was performed in triplicate for each biological replicate. Total tea polyphenol content was determined using the ferrous tartrate colorimetric method based on GB/T 8313-2002. Another 0.1 mL of the supernatant was mixed with 0.9 mL of ultrapure water and 1 mL of ferrous tartrate reagent, followed by the addition of 3 mL of pH 7.5 phosphate buffer in triplicate. After thorough mixing, the absorbance was measured at 540 nm. The assay was performed in triplicate for each biological replicate. Total tea polyphenol content was calculated from the absorbance according to GB/T 8313-2002.

### 2.4. Determination of Caffeine Content

Caffeine content was determined according to the method described by Ge et al. [23], which was based on the Chinese national standard GB/T 8312–2013. Three 0.1-g tea powder samples (biological replicates) were each extracted using 5 mL of boiling distilled water in a 100 °C water bath for 45 min, and the mixtures were centrifuged at 12,000 rpm for 10 min at 4 °C. Then, 300 μL of the supernatant was mixed with 320 μL of 0.01 M HCl and 80 μL of 50% basic lead acetate solution, diluted to 8 mL with distilled water, and centrifuged. Thereafter, 4 mL of the supernatant was mixed with 16 μL of 4.5 M H_2_SO_4_, diluted to 8 mL, and centrifuged again. Absorbance was measured at 274 nm. Caffeine concentrations in the tea samples were then calculated against this validated authentic caffeine reference standard curve (y = 0.0505x − 0.0041 R^2^ = 0.9994).

### 2.5. Zebrafish Husbandry and Embryo Collection

All procedures involving zebrafish were performed in accordance with institutional guidelines for animal care and use and were approved by the Institutional Animal Care and Use Committee (IACUC) of Baihuangouke Biotechnology Co., Ltd., Guangzhou, China (Approval No. BH2508140-3). Wild-type adult AB zebrafish were maintained in a recirculating aquaculture system in 3.0-L tanks at a stocking density of 5 fish/L, with dissolved oxygen and water quality maintained under stable conditions. The water temperature was maintained at 28 ± 1 °C, and the pH was maintained at 7.0–7.5, under a 14 h light/10 h dark photoperiod. They were fed three times daily at fixed intervals, including brine shrimp (Aquamaster, Binzhou, China) twice daily and commercial fish feed (Tetra, Melle, Germany) once daily.

For embryo collection, sexually mature male and female zebrafish were paired at a 1:1 ratio with an overnight separating barrier. To initiate natural spawning, the divider was removed the following morning within 1 h post-onset of light. Fertilized embryos were collected and maintained in 1× E3 medium containing 5 mM NaCl, 0.17 mM KCl, 0.33 mM CaCl_2_·2H_2_O, and 0.33 mM MgSO_4_·7H_2_O (supplemented with 0.3 ppm methylene blue as an antifungal agent) in 10-cm Petri dishes at 28.5 °C under the same photoperiod until experimental treatment.

### 2.6. Detection of L-Theanine MTC in Zebrafish

Wild-type AB zebrafish larvae at 4 days post-fertilization (dpf) were randomly distributed to 6-well plates. Then, 30 zebrafish larvae were used for the maximum tolerated concentration (MTC) assessment of L-theanine. The larvae were subsequently exposed to different concentrations of L-theanine (Aladdin, Shanghai, China) according to the experimental design shown in Table S1. After a 24-h incubation window, mortality and morphological abnormalities were systematically recorded to determine the maximum tolerated concentration (MTC) of L-theanine (Figure S1 and Table S1).

### 2.7. Experimental Grouping and Establishment of the PTZ-Induced Hyperactivity Model

To minimize animal use, the sample size was determined based on previous studies using PTZ-induced zebrafish models and preliminary experimental observations [24]. Zebrafish larvae were selected to assess behavioral responses that cannot be fully reproduced in vitro. Treatment concentrations were determined through preliminary maximum tolerated concentration (MTC) assessments to minimize potential adverse effects.

At 5 dpf, wild-type AB zebrafish larvae were randomly transferred to 6-well plates (30 larvae per well). The experimental groups included an untreated control group, a PTZ-treated model group [25,26], a positive-control group treated with 125 μg/mL melatonin (Aladdin, Shanghai, China), and the different sample-treatment groups. The treatment concentrations used in each group are listed in Table S2.

Larvae within the positive control and sample-treatment groups were pre-treated with the corresponding compounds or tea infusions for 24 h, whereas the control and model groups were maintained concurrently under the same conditions without sample treatment. After pre-treatment, the larvae were rinsed with PBS to remove extracellular residue. All groups except the untreated control group were subsequently exposed to 5 mM PTZ (Shanghai Yihe Biotechnology Co., Ltd., Shanghai, China) to induce hyperactivity. During PTZ exposure, zebrafish larvae were continuously monitored. Humane endpoints included severe or persistent abnormal swimming, abnormal sinking, or floating, and affected larvae were promptly euthanized. At the end of the experiment or upon reaching a humane endpoint, larvae were immersed in ice-chilled water (0–4 °C) for at least 20 min, and cessation of heartbeat was confirmed by microscopic examination. Subsequently, the larvae were transferred to prechilled microcentrifuge tubes and frozen at −80 °C for at least 30 min to ensure irreversible death.

### 2.8. Zebrafish Behavioral Assessment

Briefly, 0.3 g tea samples of BY and LJ43 from Section 2.1 were infused with 30 mL of boiling water for 5 min. After filtration, a tea infusion stock solution with a dry-tea input equivalent of 10 mg/mL was obtained, which was then diluted 1:10 to 1000 μg/mL for zebrafish treatment. Following the treatments outlined in Section 2.7, zebrafish larvae were individually transferred to 96-well plates (one larva per well) (Nest Biotech, Wuxi, Jiangsu, China) containing 200 μL of the corresponding solution. Behavioral responses were recorded for 1 h using a high-throughput 2D behavioral analysis system (BHW-Z-2D; Baihuan Biotechnology Co., Ltd., Guangzhou, China), which automatically quantified locomotor activity without subjective assessment. Ten zebrafish larvae were analyzed per group for each behavioral parameter. We assessed the regulatory effects of the different treatments on PTZ-induced hyperactivity, as reported previously [27,28]. High-speed movement distance was defined as the cumulative distance traveled during high-speed movement (>20 mm/s), whereas high-speed movement duration was defined as the cumulative time engaged in high-speed movement (>20 mm/s). These parameters were used to assess PTZ-induced hyperactivity and were utilized to evaluate the hyperactivity alleviation effects of the tea samples and individual bioactive compounds.

### 2.9. Quantification of Receptor mRNA Levels

Following the treatments described in Section 2.7, three biological replicates were prepared for each group, with 30 zebrafish larvae pooled per replicate for RNA extraction and quantitative real-time PCR analysis. Total RNA was extracted using an RNA extraction kit (Lot No. 7E2140C5; Vazyme Biotech Co., Ltd., Nanjing, China). RNA concentration was measured by NanoPhotometer N60 (IMPLEN, Munich, Germany), and equal amounts of RNA were used for reverse transcription with a reverse transcription kit (Lot No. 7E0650B4; Vazyme Biotech Co., Ltd., Nanjing, China) to ensure consistent cDNA input across samples. To evaluate the regulatory impacts on *gabra1* and *mtnr1aa*, the relative expression levels of *gabra1* and *mtnr1aa* were determined using quantitative real-time PCR (qRT-PCR) (CFX Connect, Bio-Rad, Hercules, CA, USA). The primer sequences used in this study are shown in Table S3. Relative expression levels were calculated using the standard comparative cycle threshold (2^−∆∆CT^) method, normalized against *β-actin* expression.

### 2.10. Molecular Docking

The primary amino acid sequences of zebrafish GABRA1 (UniProt ID: Q08BJ3) and MTNR1AA (UniProt ID: P51046) were retrieved from the UniProt database (https://www.uniprot.org/ (accessed on 24 April 2026)). The corresponding 3D protein structures of GABRA1 and MTNR1AA were obtained from the AlphaFold Protein Structure Database (https://alphafold.com/). Structural files for GABA (CAS 56-12-2), melatonin (CAS 73-31-4), and L-theanine (CAS 3081-61-6) ligands were downloaded in SDF format from the PubChem database (https://pubchem.ncbi.nlm.nih.gov/ (accessed on 24 April 2026)). The ligand SDF format files were converted to PDB format using PyMOL v 3.1.6.1. For receptor preparation, AutoDockTools 1.5.7 was used to remove water molecules, add polar hydrogen atoms, and compute Gasteiger charges for the target proteins. Molecular docking simulations were executed using AutoDock Vina. Finally, PyMOL and LigPlot+ were employed to visualize and analyze the docking results [29].

### 2.11. Statistical Analysis

Data were analyzed using SPSS 27.0 software (IBM Ltd., Corp., Armonk, NY, USA) and presented as the mean ± standard deviation (SD). Comparisons between two groups were conducted using unpaired *t*-tests. For analyses involving multiple groups, including the control, model, positive control, and each treatment group, one-way ANOVA analysis was deployed, followed by Tukey’s multiple comparison test. Statistical significance was established at *p* < 0.05 across all analyses.

## 3. Results

### 3.1. Quality-Related Components of BY and LJ43

**Figure 1:**
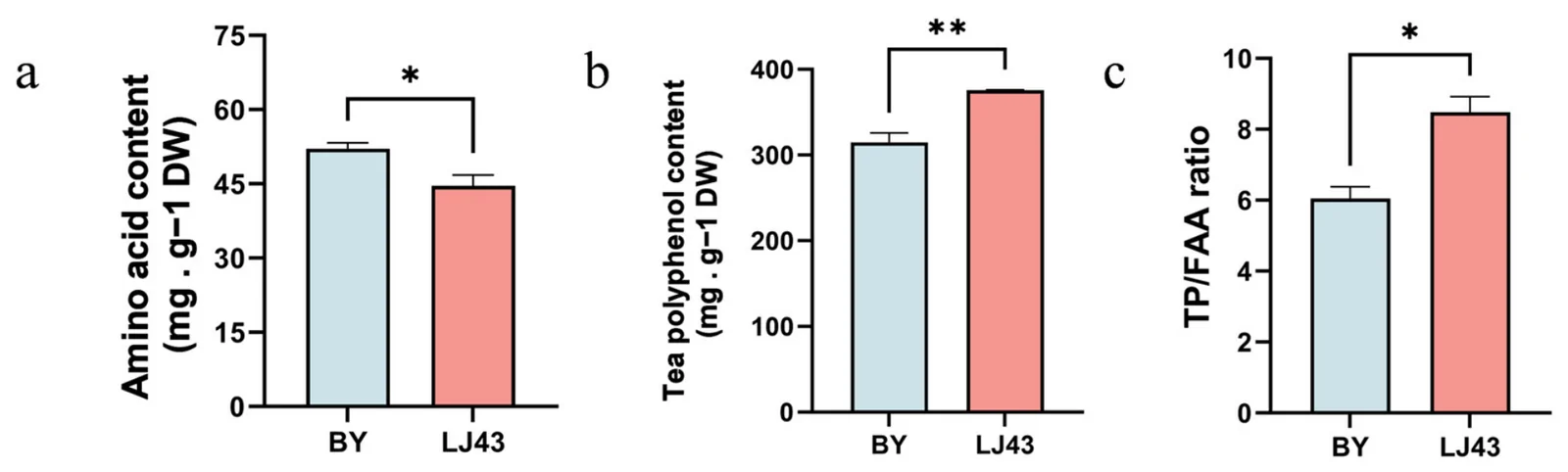
Comparative analysis of basic biochemical traits between ‘Baiye 1’ (BY) and ‘Longjing 43’ (LJ43) tea. (**a**) Total free amino acid content; (**b**) tea polyphenol content; (**c**) TP/FAA ratio. Data are presented as mean ± SD (*n* = 3). Asterisks indicate significant differences between BY and LJ43 (* *p* < 0.05, ** *p* < 0.01).

Based on preliminary tests revealing divergent L-theanine and caffeine profiles, BY and LJ43 were selected as representative models to describe different metabolic characteristics. Quantitative profiling of major bioactive components was performed to establish the basic biochemical characteristics (Figure 1). The total free amino acid (FAA) content of BY was significantly higher than that of LJ43, yielding values of 52.11 ± 2.06 mg·g^−1^ DW and 44.58 ± 3.84 mg·g^−1^ DW, respectively (Figure 1a). In contrast, LJ43 possessed significantly higher tea polyphenol (TP) content than BY, with values of 375.92 ± 0.23 mg·g^−1^ DW and 314.69 ± 19.18 mg·g^−1^ DW, respectively (Figure 1b). The TP/FAA ratio of LJ43 (8.48 ± 0.77) was also notably higher than that of BY (6.05 ± 0.57) (Figure 1c).

### 3.2. Metabolomic Profiling of BY and LJ43

**Figure 2:**
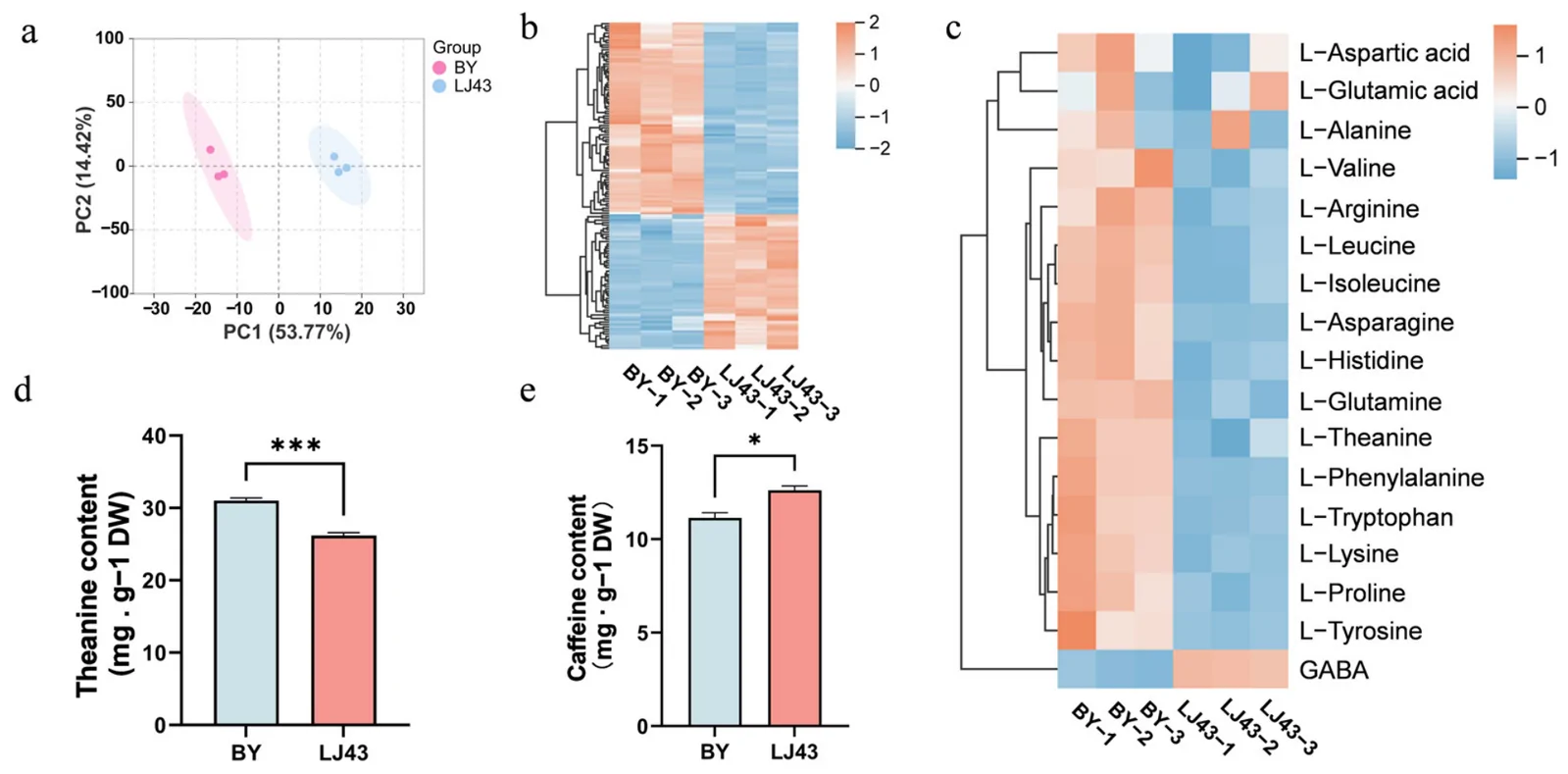
Metabolomic profiling and differential accumulation patterns of quality-related metabolites in BY and LJ43. (**a**) Principal component analysis (PCA) score plot of metabolite profiles from BY and LJ43 samples. (**b**) Hierarchical clustering heatmap of differential metabolites detected between BY and LJ43. (**c**) Hierarchical clustering heatmap of amino acids. (**d**) L-theanine content; (**e**) caffeine content. Data are presented as mean ± SD (*n* = 3) in (**d**,**e**). Asterisks indicate significant differences between BY and LJ43 (* *p* < 0.05, *** *p* < 0.001). In the heatmaps, metabolite abundances were normalized by *Z*-score transformation. Red and blue indicate relatively high and low accumulation levels, respectively.

To characterize the compositional differences between BY and LJ43, untargeted metabolomic analysis was performed. The resulting PCA showed that the biological replicates clustered closely within each group, with distinct clustering of the two tea cultivars in the PCA score plot (Figure 2a). Hierarchical clustering analysis further indicated that the metabolites exhibited different accumulation patterns between the two samples (Figure 2b).

To further characterize the underlying amino acid profile, the distribution of individual free amino acids and their derivatives was systematically examined. Most amino acids accumulated at higher levels in BY than in LJ43, with L-theanine showing a particularly pronounced difference. Conversely, GABA accumulation was higher in LJ43 than in BY, demonstrating that the accumulation patterns varied among individual amino acids (Figure 2c). Independent targeted quantification of prime bioactives confirmed that BY contained significantly more L-theanine, whereas LJ43 was dominated by elevated caffeine (Figure 2d,e). Collectively, the two cultivars exhibited markedly different baseline levels of key bioactive compounds.

### 3.3. Effects of BY and LJ43 on PTZ-Induced Hyperactivity

**Figure 3:**
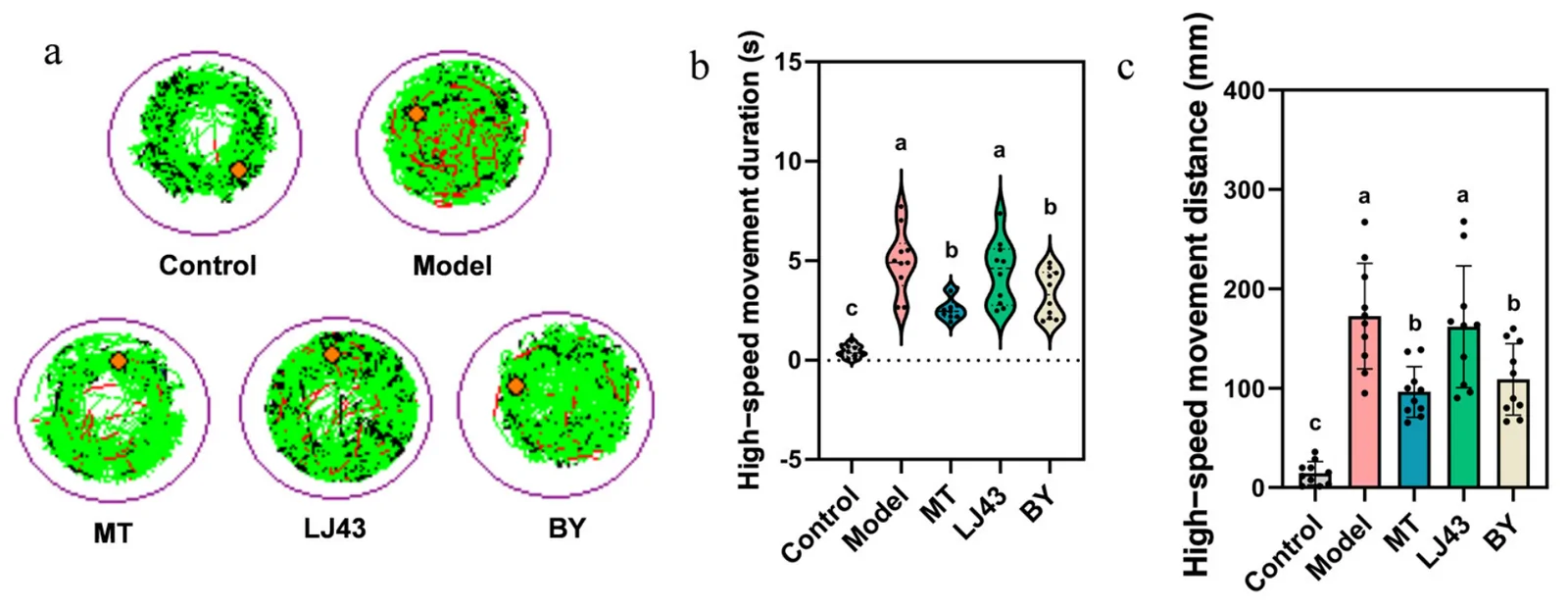
Effects of tea infusion from BY and LJ43 on PTZ-induced hyperactivity in zebrafish larvae. The concentration of tea infusion was 1000 μg/mL. (**a**) Representative locomotor trajectory plots of zebrafish larvae in different treatment groups. (**b**) High-speed movement duration. Data are shown as individual data points with violin plots. Different lowercase letters indicate significant differences among groups at *p* < 0.05. (**c**) High-speed movement distance. Data are presented as mean ± SD (*n* = 10). Control, untreated larvae; model, PTZ-treated larvae; MT, melatonin-treated positive control; LJ43, larvae treated with tea infusion prepared from LJ43; BY, larvae treated with tea infusion prepared from BY.

To verify the functional differences revealed by metabolomic analysis, behavioral assays were carried out on PTZ-induced AB strain larvae. The results showed increased locomotor trajectories in the model group (Figure 3a). Quantitative analysis of behavioral data showed that high-speed movement duration and high-speed movement distance were significantly elevated following PTZ administration, indicating the zebrafish PTZ-induced hyperactivity model was successfully established (Figure 3b,c). Following treatment with tea infusions prepared from LJ43 or BY, these hyperactivity phenotypes were suppressed. However, only the BY-treated group exhibited statistically significant reductions compared with the model group. These findings reveal that BY was more effective in attenuating PTZ-induced hyperactivity than LJ43, while remaining less potent than the reference standard melatonin (Figure 3b,c).

### 3.4. Effects of L-Theanine and Its Combinations on PTZ-Induced Hyperactivity

**Figure 4:**
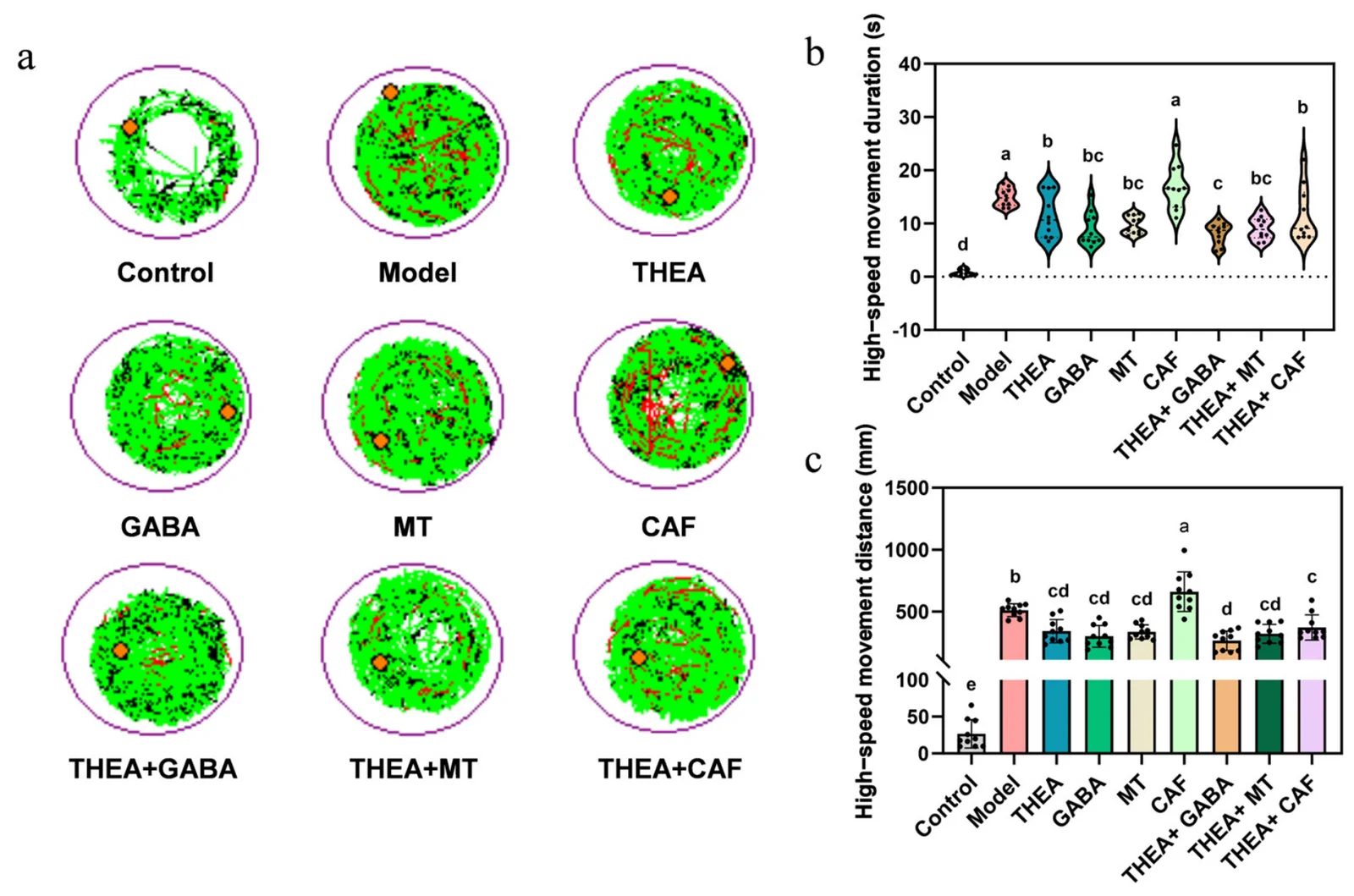
Effects of L-theanine or in combination with GABA, melatonin, or caffeine on PTZ-induced hyperactivity in zebrafish larvae. (**a**) Representative locomotor trajectories of zebrafish larvae in different treatment groups. (**b**) High-speed movement duration, shown as violin plots with individual data points. Different lowercase letters indicate significant differences among groups at *p* < 0.05. (**c**) High-speed movement distance. Data are presented as mean ± SD (*n* = 10). Control, untreated larvae; Model, PTZ-treated larvae; THEA, L-theanine-treated larvae; GABA, γ-aminobutyric acid-treated larvae; MT, melatonin-treated larvae; CAF, caffeine-treated larvae; THEA + GABA, larvae treated with L-theanine and GABA; THEA + MT, larvae treated with L-theanine and melatonin; THEA + CAF, larvae treated with L-theanine and caffeine. Each dot represents one larva.

Acute toxicity screening established that the maximum tolerated concentration (MTC) of L-theanine at thresholds of 2000 μg/mL caused no obvious abnormal phenotypes or mortality in zebrafish larvae (Figure S1a,b). Utilizing this non-toxic baseline within the PTZ-induced hyperactivity model, we systematically compared the effects of L-theanine alone and in combination with GABA, melatonin, or caffeine on PTZ-induced hyperactivity (Figure 4). Behavioral tracking revealed that all treatment groups alleviated PTZ-triggered hyperactivity to varying degrees, except for those subjected to caffeine treatment. Quantitative analysis of high-speed movement duration and high-speed movement distance showed consistent trends with the behavioral observations (Figure 4b,c).

Compared with the model group, individual L-theanine application significantly decreased high-speed movement duration and high-speed movement distance in hyperactive zebrafish, demonstrating its hyperactivity attenuation. Furthermore, combinations of L-theanine with either GABA or melatonin outperformed L-theanine monotherapy. The THEA + MT group showed a trend toward greater attenuation of PTZ-induced hyperactivity, while the THEA + GABA group showed a more pronounced effect (Figure 4b,c). Although caffeine independently aggravated the hyperactive state, the co-treatment of L-theanine and caffeine reduced PTZ-induced hyperactivity compared with caffeine treatment alone (Figure 4b,c).

### 3.5. Effects of the Gene Expression of gabra1 and mtnr1aa

**Figure 5:**
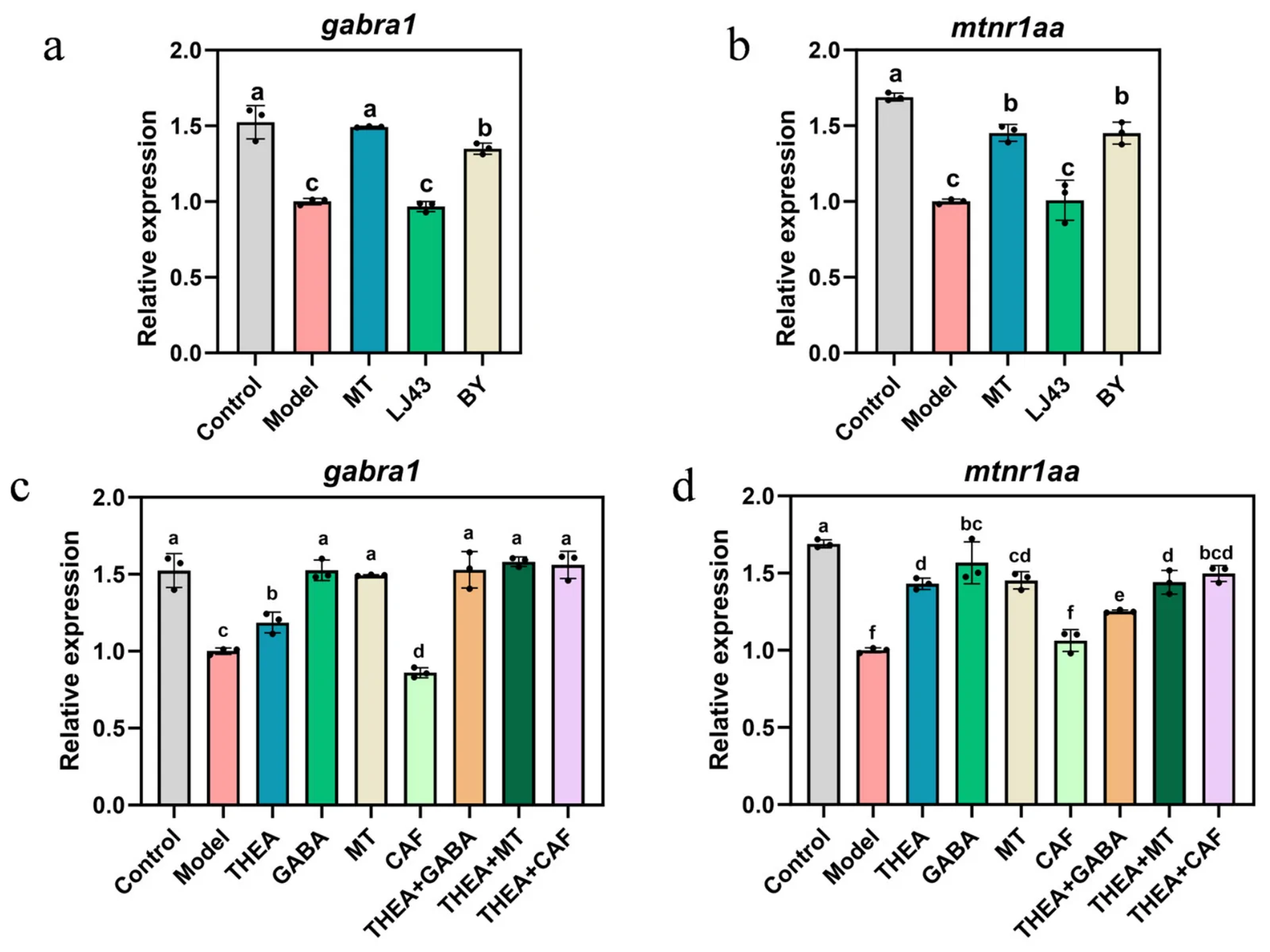
Effects of tea infusion and key functional components on the expression of *gabra1* and *mtnr1aa* in zebrafish larvae. (**a**) Relative expression of *gabra1* in the Control, Model, MT, LJ43, and BY groups. (**b**) Relative expression of *mtnr1aa* in the Control, Model, MT, LJ43, and BY groups. (**c**) Relative expression of *gabra1* in the Control, Model, THEA, GABA, MT, CAF, THEA + GABA, THEA + MT, and THEA + CAF groups. (**d**) Relative expression of *mtnr1aa* in the Control, Model, THEA, GABA, MT, CAF, THEA + GABA, THEA + MT, and THEA + CAF groups. Control, untreated larvae; Model, PTZ-treated larvae; MT, melatonin-treated larvae; LJ43, larvae treated with tea infusion prepared from LJ43; BY, larvae treated with tea infusion prepared from BY; THEA, L-theanine-treated larvae; GABA, γ-aminobutyric acid-treated larvae; CAF, caffeine-treated larvae; THEA + GABA, larvae treated with L-theanine and GABA; THEA + MT, larvae treated with L-theanine and melatonin; THEA + CAF, larvae treated with L-theanine and caffeine. Data are presented as mean ± SD (*n* = 3). Different lowercase letters indicate significant differences among groups at *p* < 0.05.

To investigate the effects of BY, LJ43, and their bioactive components on PTZ-induced hyperactivity, qRT-PCR was used to detect the expression levels of *gabra1* and *mtnr1aa* in PTZ-induced hyperactivity zebrafish (Figure 5). The results revealed that compared with the model group, BY significantly upregulated the expression of *gabra1* and *mtnr1aa*, while no significant changes were observed in the LJ43 treatment group. Similarly, the melatonin group exhibited a marked upregulation in *gabra1* and *mtnr1aa* as well (Figure 5a,b). These results were basically consistent with the previous behavioral analysis and the behavioral differences observed between BY and LJ43.

Further analysis of the effects of L-theanine and its combinations on the expression of *gabra1* and *mtnr1aa* revealed significant differences in transcript levels among groups. While L-theanine significantly upregulated the expression of these two genes, its effect was weaker than that of GABA or melatonin treatments. Combinations of L-theanine with GABA or melatonin induced higher expression levels of *gabra1* and *mtnr1aa* than L-theanine monotherapy (Figure 5c,d). These results indicate that the effects on PTZ-induced hyperactivity of L-theanine and its co-treatments are potentially associated with altered expression of GABA- and melatonin-related receptor genes.

### 3.6. Molecular Docking of GABRA1 and MTNR1AA

**Figure 6:**
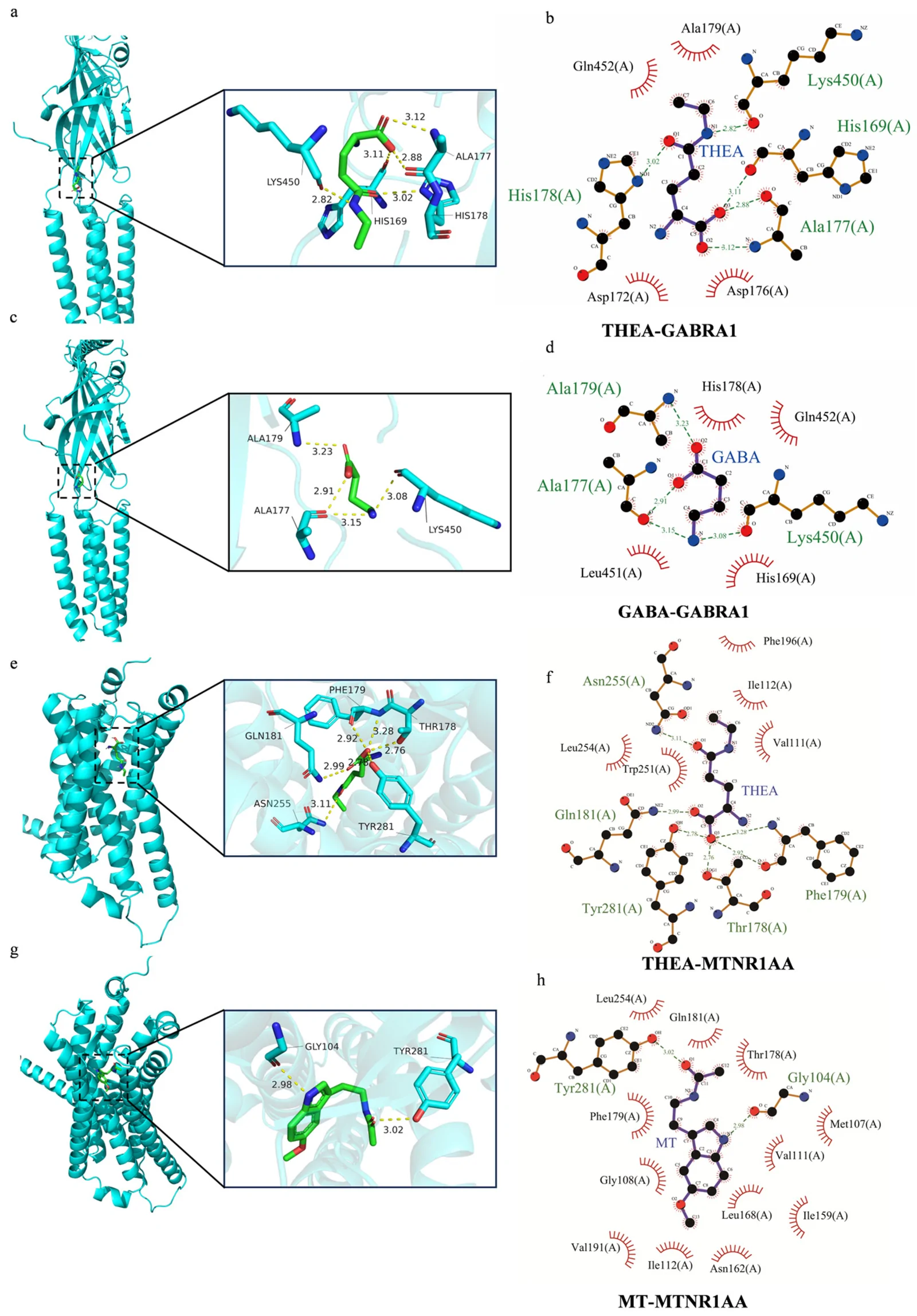
Molecular docking of GABA, THEA and MT with the GABRA1 and MTNR1AA receptors. 3D and 2D predicted binding poses of THEA-GABRA1 (**a**,**b**), GABA-GABRA1 (**c**,**d**), THEA-MTNR1AA (**e**,**f**) and MT-MTNR1AA (**g**,**h**). Yellow dashed lines represent putative hydrogen-bond interactions, with distances labeled in Å. The ligand is shown in green (carbon atoms), red (oxygen atoms), and dark blue (nitrogen atoms). The protein residues are shown in cyan (carbon atoms), with oxygen and nitrogen atoms colored as described above. The labels indicate the specific amino acid residues involved in the binding site.

To map the structural binding mode of L-theanine, molecular docking simulations were conducted against the target receptors GABRA1 and MTNR1AA. The results showed that L-theanine may interact with the amino acid residues of GABRA1 via hydrogen bonds and hydrophobic forces, with a binding affinity of −5.5 kcal/mol. In this binding mode, L-theanine was predicted to form hydrogen-bond interactions with GABRA1 residues HIS169, ALA177, HIS178, and LYS450 (Figure 6a,b), exhibiting a similar binding profile to the endogenous ligand GABA (Figure 6c,d). L-theanine docking simulations predicted a putative binding pose of L-theanine near MTNR1AA residues THR178, PHE179, and GLN181, suggesting potential interactions with the receptor structure (Figure 6e,f). The predicted docking score for the L-theanine–MTNR1AA complex was −5.7 kcal/mol, suggesting potential structural compatibility between L-theanine and the receptor model (Figure 6e,f). For comparison, the predicted binding mode of melatonin with MTNR1AA is shown in Figure 6g,h. The respective predicted affinities are summarized in Table S4. Additionally, the predicted docking pose of L-theanine with the human pentameric GABA_A receptor is presented in Figure S2. Collectively, L-theanine was predicted to exhibit moderate binding affinities toward both GABRA1 and MTNR1AA, providing a computational hypothesis for its potential interactions with these proteins.

## 4. Discussion

Tea contains diverse bioactive compounds, including amino acids, polyphenols, and alkaloids, and these compounds contribute to various physiological functions [30]. In this study, comparative metabolomics and biochemical profiling revealed marked chemical differences between cultivars BY and LJ43. BY contained higher levels of L-theanine and lower levels of caffeine than LJ43. When deployed in an in vivo PTZ-induced zebrafish hyperactivity model, these distinct metabolic signatures translated into disparate behavioral outcomes, with BY showing a greater attenuation of PTZ-induced hyperactivity than LJ43. The expression of *gabra1* and *mtnr1aa* was further analyzed to explore the potential pathways involved in these effects. L-theanine alone also alleviated the hyperactivity phenotype, while combination treatments produced stronger effects for selected behavioral endpoints. Notably, the concentration of L-theanine used in the monotherapy assay was substantially higher than its levels in the tea infusions. However, these findings support the potential of L-theanine to attenuate PTZ-induced hyperactivity, while the behavioral differences between BY and LJ43 may reflect the combined effects of multiple bioactive constituents.

The functional differences among various teas are closely associated with the composition of their bioactive constituents [31]. Prior investigations highlight that L-theanine and caffeine are crucial components affecting the neurological functions of tea, and variations in their contents and ratios can lead to divergent functional performances [18,32,33]. BY contained higher levels of L-theanine and lower levels of caffeine, along with differences in other bioactive constituents. These compositional differences may collectively contribute to the distinct behavioral effects of the two tea infusions. Importantly, the L-theanine monotherapy assay demonstrated that L-theanine significantly attenuated PTZ-induced hyperactivity, supporting its potential as a bioactive compound for alleviating hyperactivity. On this basis, L-theanine was considered a plausible contributor to the observed behavioral effects. However, because BY and LJ43 differ in multiple bioactive constituents, the specific contribution of L-theanine to their distinct behavioral effects remains difficult to determine.

Although the function of L-theanine has been reported in several mammalian and clinical studies, empirical validation within aquatic vertebrate models remains limited [28,34]. Here, L-theanine treatment reduced the high-speed movement distance and high-speed movement duration in hyperactive zebrafish larvae, thereby supporting its ameliorative effect on PTZ-induced hyperactivity. Furthermore, co-treatment of L-theanine with GABA or melatonin yielded more significant improvements in behavioral phenotypes and enhanced upregulation of GABA- and melatonin-related receptor genes than L-theanine monotherapy. These results suggest that the combination of GABA and L-theanine may be more effective in alleviating PTZ-induced hyperactivity than L-theanine alone, which is consistent with previous findings reporting enhanced effects of combined GABA and L-theanine treatment [35]. They also highlight the potential application of L-theanine-based combinations in functional food development.

In contrast, individual caffeine treatment exacerbated the PTZ-induced hyperactivity in zebrafish, which is consistent with its established role as a central nervous system stimulant [14]. At the specified doses used in this study, the combination of L-theanine and caffeine partially reversed caffeine-induced effects compared with caffeine treatment alone, indicating that L-theanine may attenuate caffeine-induced hyperarousal. However, these results do not exclude the possibility that L-theanine and caffeine may exhibit antagonistic interactions under other dose or ratio conditions. Therefore, their interaction should be further clarified through systematic experiments involving broader dose gradients and different combination ratios, as the biological effects of combined bioactive compounds may vary substantially depending on their relative proportions [36,37].

At the molecular level, the expression levels of the zebrafish GABAergic and melatonin-related receptor genes *gabra1* and *mtnr1aa* were significantly upregulated by both BY and L-theanine. This transcription profile was further amplified following the co-administration of L-theanine with GABA or melatonin, and this expression pattern was generally consistent with the behavioral results. These findings suggest that GABA- and melatonin-related signaling pathways may be involved in modulation of PTZ-induced hyperactivity, which are associated with inhibitory neurotransmission and circadian regulation, respectively [38,39]. Molecular docking simulations predicted possible interactions between L-theanine and the corresponding receptors. Since our current data are mainly derived from phenotypic assays, gene expression analysis, and computational simulations, they remain insufficient to establish a definitive molecular mechanism. Consequently, further studies involving receptor antagonists, genetic manipulation, protein-level validation, and direct binding assays are required to confirm the proposed mechanisms.

## 5. Conclusions

This study revealed distinct metabolic profiles between BY and LJ43 tea cultivars, with BY exhibiting higher L-theanine and lower caffeine contents. In the PTZ-induced zebrafish model, BY tea infusion significantly attenuated hyperactivity, whereas LJ43 showed a comparatively limited effect. Importantly, L-theanine treatment significantly reduced PTZ-induced hyperactivity, supporting its potential as a bioactive compound for alleviating excessive locomotor activity. The effects of combining L-theanine with GABA or melatonin showed greater reductions in high-speed movement distance and duration. Furthermore, BY and L-theanine treatments were associated with changes in the expression of *gabra1* and *mtnr1aa*. Molecular docking analyses provided preliminary computational predictions of potential interactions between L-theanine and GABRA1 or MTNR1AA. Overall, these findings highlight L-theanine as a candidate bioactive compound for attenuating PTZ-induced hyperactivity and provide insights into the potential behavioral effects of tea-derived constituents. These findings reflect acute locomotor responses, as validated sleep parameters were not measured in this study. Moreover, although BY and LJ43 exhibited distinct metabolic profiles and behavioral effects, the contribution of L-theanine to their cultivar-specific behavioral differences remains to be clarified.

## Data Availability

The original contributions presented in this study are included in the article/Supplementary Material. Further inquiries can be directed to the corresponding authors.

## Supplementary Materials

The following supporting information can be downloaded at https://www.mdpi.com/article/10.3390/foods15213992/s1, Figure S1: Determination of the maximum tolerated concentration (MTC) of L-theanine (THEA) in zebrafish larvae. Figure S2: Molecular docking of L-theanine with the human pentameric GABA_A receptor (PDB: 9FG7). The overall receptor structure and enlarged view of the predicted ligand-binding pocket. Yellow dashed lines indicate predicted hydrogen bonds. The predicted binding affinity was −6.2 kcal/mol, with distances labeled in Å. The ligand is shown in green (carbon atoms), red (oxygen atoms), and dark blue (nitrogen atoms). The protein residues are shown in cyan (carbon atoms), with oxygen and nitrogen atoms colored as described above. The labels indicate the specific amino acid residues involved in the binding site. Table S1. Results of maximum tolerated concentration (MTC) exploration for THEA (*n* = 30). Table S2. Concentrations of treatment groups. Table S3. Primer sequences for *gabra1* and *mtnr1aa* in zebrafish used for qRT-PCR. Table S4. Molecular docking binding affinities of GABA, MT and THEA with GABRA1 and MTNR1AA.

## References

[1] Jones B.E. (2020). Arousal and Sleep Circuits. Neuropsychopharmacol.

[2] Riemann D., Spiegelhalder K., Feige B., Voderholzer U., Berger M., Perlis M., Nissen C. (2010). The Hyperarousal Model of Insomnia: A Review of the Concept and Its Evidence. Sleep. Med. Rev..

[3] Li J., Cao D., Huang Y., Chen Z., Wang R., Dong Q., Wei Q., Liu L. (2022). Sleep Duration and Health Outcomes: An Umbrella Review. Sleep. Breath..

[4] De Crescenzo F., D’Alò G.L., Ostinelli E.G., Ciabattini M., Di Franco V., Watanabe N., Kurtulmus A., Tomlinson A., Mitrova Z., Foti F. (2022). Comparative Effects of Pharmacological Interventions for the Acute and Long-Term Management of Insomnia Disorder in Adults: A Systematic Review and Network Meta-Analysis. Lancet.

[5] Le J., Li L., Wang C., Hu Q., Shao K., Zhao Y., Jin Y. (2025). The Function and Application Potential of L-Theanine: From Biotechnological Production, Biosynthesis, Bioactivity to Food Industry. Food Chem..

[6] Unno K., Noda S., Kawasaki Y., Yamada H., Morita A., Iguchi K., Nakamura Y. (2017). Reduced Stress and Improved Sleep Quality Caused by Green Tea Are Associated with a Reduced Caffeine Content. Nutrients.

[7] Willemse D., Rado M., Lilly M. (2026). Contribution of Proteomics and Metabolomics Data to Understanding of Health Benefits of Tea. Chin. Herb. Med..

[8] Moulin M., Crowley D.C., Xiong L., Guthrie N., Lewis E.D. (2024). Safety and Efficacy of AlphaWave^®^ L-Theanine Supplementation for 28 Days in Healthy Adults with Moderate Stress: A Randomized, Double-Blind, Placebo-Controlled Trial. Neurol. Ther..

[9] Bulman A., D’Cunha N.M., Marx W., Turner M., McKune A., Naumovski N. (2025). The Effects of L-Theanine Consumption on Sleep Outcomes: A Systematic Review and Meta-Analysis. Sleep. Med. Rev..

[10] Gardiner C., Weakley J., Burke L.M., Roach G.D., Sargent C., Maniar N., Townshend A., Halson S.L. (2023). The Effect of Caffeine on Subsequent Sleep: A Systematic Review and Meta-Analysis. Sleep. Med. Rev..

[11] Di X., Yan J., Zhao Y., Zhang J., Shi Z., Chang Y., Zhao B. (2010). L-Theanine Protects the APP (Swedish Mutation) Transgenic SH-SY5Y Cell against Glutamate-Induced Excitotoxicity via Inhibition of the NMDA Receptor Pathway. Neuroscience.

[12] Kakuda T., Nozawa A., Sugimoto A., Niion H. (2002). Inhibition by Theanine of Binding of [3H]AMPA, [3H]Kainate, and [3H]MDL 105,519 to Glutamate Receptors. Biosci. Biotechnol. Biochem..

[13] Lin Y.-S., Lange D., Baur D.M., Foerges A., Chu C., Li C., Elmenhorst E.-M., Neumaier B., Bauer A., Aeschbach D. (2024). Repeated Caffeine Intake Suppresses Cerebral Grey Matter Responses to Chronic Sleep Restriction in an A1 Adenosine Receptor-Dependent Manner: A Double-Blind Randomized Controlled Study with PET-MRI. Sci. Rep..

[14] Reichert C.F., Deboer T., Landolt H.-P. (2022). Adenosine, Caffeine, and Sleep–Wake Regulation: State of the Science and Perspectives. J. Sleep. Res..

[15] Zhang J., Li H., Ma C., Zhang S., Wu G., Shen B., Chen B., Chen L., Zhang Q., Yang G. (2025). Distinct Effects of Tea Types and Components on Circadian Rhythm Entrainment and Clock Gene Regulation. npj Sci. Food.

[16] Yu Q., Huang C., Zhu R., Lu D., Liu L., Lai J., Zhong X., Guan J., Zhou S., Tong Y. (2023). Chemometrics-Based Investigation of Non-Volatiles/Volatiles Flavor of Tencha (*Camellia Sinensis* Cv. *Yabukita*, *Longjing 43* and *Baiye 1*). Food Res. Int..

[17] Zeng L., Fu Y.-Q., Gao Y., Wang F., Liang S., Yin J.-F., Fauconnier M.-L., Ke L., Xu Y.-Q. (2024). Dynamic Changes of Key Metabolites in Longjing Green Tea during Processing Revealed by Widely Targeted Metabolomic Profiling and Sensory Experiments. Food Chem..

[18] Payne E.R., Aceves-Martins M., Dubost J., Greyling A., de Roos B. (2025). Effects of Tea (*Camellia sinensis*) or Its Bioactive Compounds l-Theanine or l-Theanine plus Caffeine on Cognition, Sleep, and Mood in Healthy Participants: A Systematic Review and Meta-Analysis of Randomized Controlled Trials. Nutr. Rev..

[19] Khan K.M., Collier A.D., Meshalkina D.A., Kysil E.V., Khatsko S.L., Kolesnikova T., Morzherin Y.Y., Warnick J.E., Kalueff A.V., Echevarria D.J. (2017). Zebrafish Models in Neuropsychopharmacology and CNS Drug Discovery. Br. J. Pharmacol..

[20] Rihel J., Prober D.A., Arvanites A., Lam K., Zimmerman S., Jang S., Haggarty S.J., Kokel D., Rubin L.L., Peterson R.T. (2010). Zebrafish Behavioral Profiling Links Drugs to Biological Targets and Rest/Wake Regulation. Science.

[21] Parthasarathy S., Aly S.H., Tharumasivam S.V., Siva D., Krishnasamy G., Chavaan A., El-Shazly M. (2025). Zebrafish in the Spotlight: Expanding Frontiers in Toxicology and Drug Discovery. Toxicol. Res..

[22] Ge S., Zhang Z., Hu Q., Wang Q., Gong X., Huang F., Zhang L., Han W., Luo F., Li X. (2025). Metabolomics Analysis Reveals Crucial Effects of Arbuscular Mycorrhizal Fungi on the Metabolism of Quality Compounds in Shoots and Roots of *Camellia sinensis* L.. Plant Physiol. Biochem..

[23] Ge S., Wang Y., Shen K., Wang Q., Ahammed G.J., Han W., Jin Z., Li X., Shi Y. (2024). Effects of Differential Shading on Summer Tea Quality and Tea Garden Microenvironment. Plants.

[24] de Miranda F.B., Schimith L.E., da Costa Silva D.G., de Oliveira Vian C., da Luz D.B., de Aguiar R.F., da Rocha C.Y.M., Siebel A.M., Oses J.P., Hort M.A. (2025). Effects of Polydatin on Pentylenetetrazol-Induced Seizures in Zebrafish Larvae. Future Pharmacol..

[25] Barlow I.L., Mackay E., Wheater E., Goel A., Lim S., Zimmerman S., Woods I., Prober D.A., Rihel J. (2023). The Zebrafish Mutant Dreammist Implicates Sodium Homeostasis in Sleep Regulation. eLife.

[26] Reichert S., Pavón Arocas O., Rihel J. (2019). The Neuropeptide Galanin Is Required for Homeostatic Rebound Sleep Following Increased Neuronal Activity. Neuron.

[27] Lee D.A., Oikonomou G., Prober D.A. (2022). Large-Scale Analysis of Sleep in Zebrafish. Bio-Protocol.

[28] Zhao B., Jia X., Feng H., Tang C., Huang Y., Zhao Z., Hao J., Li H., Du J., Liu Y. (2023). Nutrient Combinations Exhibit Universal Antianxiety, Antioxidant, Neuro-Protecting, and Memory-Improving Activities. Front. Nutr..

[29] Laskowski R.A., Swindells M.B. (2011). LigPlot+: Multiple Ligand–Protein Interaction Diagrams for Drug Discovery. J. Chem. Inf. Model..

[30] Luo Q., Luo L., Zhao J., Wang Y., Luo H. (2024). Biological Potential and Mechanisms of Tea’s Bioactive Compounds: An Updated Review. J. Adv. Res..

[31] Gonçalves Bortolini D., Windson Isidoro Haminiuk C., Cristina Pedro A., de Andrade Arruda Fernandes I., Maria Maciel G. (2021). Processing, Chemical Signature and Food Industry Applications of *Camellia sinensis* Teas: An Overview. Food Chem. X.

[32] Baba Y., Takihara T., Okamura N. (2023). Theanine Maintains Sleep Quality in Healthy Young Women by Suppressing the Increase in Caffeine-Induced Wakefulness after Sleep Onset. Food Funct..

[33] Owen G.N., Parnell H., De Bruin E.A., Rycroft J.A. (2008). The Combined Effects of L-Theanine and Caffeine on Cognitive Performance and Mood. Nutr. Neurosci..

[34] Gong J., Jia X., Wang L., Lu Y., Guo R., Gong R., Hao J., Zhao Z., Duan S., Liu X. (2026). Walnut Peptide and Theanine Combination Ameliorates Sleep Disorders: A Multi-Species Study Including a Human Trial. Food Funct..

[35] Kim S., Jo K., Hong K.-B., Han S.H., Suh H.J. (2019). GABA and L-Theanine Mixture Decreases Sleep Latency and Improves NREM Sleep. Pharm. Biol..

[36] Joshi T., Deepa P.R., Sharma P.K. (2022). Effect of Different Proportions of Phenolics on Antioxidant Potential: Pointers for Bioactive Synergy/Antagonism in Foods and Nutraceuticals. Proc. Natl. Acad. Sci. India Sect. B Biol. Sci..

[37] Unno K., Taguchi K., Matsuda T., Nakamura Y. (2024). Stress-Relieving Effects of Green Tea Depend on the Ratio of Its Special Ingredients and the Infusion Conditions. Molecules.

[38] Liu J., Clough S.J., Hutchinson A.J., Adamah-Biassi E.B., Popovska-Gorevski M., Dubocovich M.L. (2016). MT1 and MT2 Melatonin Receptors: A Therapeutic Perspective. Annu. Rev. Pharmacol. Toxicol..

[39] Oishi Y., Saito Y.C., Sakurai T. (2023). GABAergic Modulation of Sleep-Wake States. Pharmacol. Ther..

