# Supplementary material for: Comparative Metabolomics Reveals L-Theanine as a Candidate Contributor to Cultivar-Specific Differences in PTZ-Induced Hyperactivity in Zebrafish
Source: Foods. 2026 Nov 7;15(21):3992. doi: 10.3390/foods15213992 (PMC13762767; doi:10.3390/foods15213992)
Supplement: Supplementary file 1 [file foods-15-03992-s001.zip › foods-4635049-supplementary.pdf]

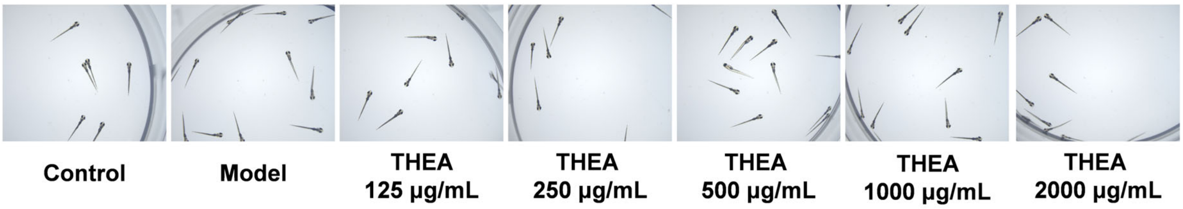

Figure S1. Determination of the maximum tolerated concentration (MTC) of L-theanine (THEA) in zebrafish larvae.

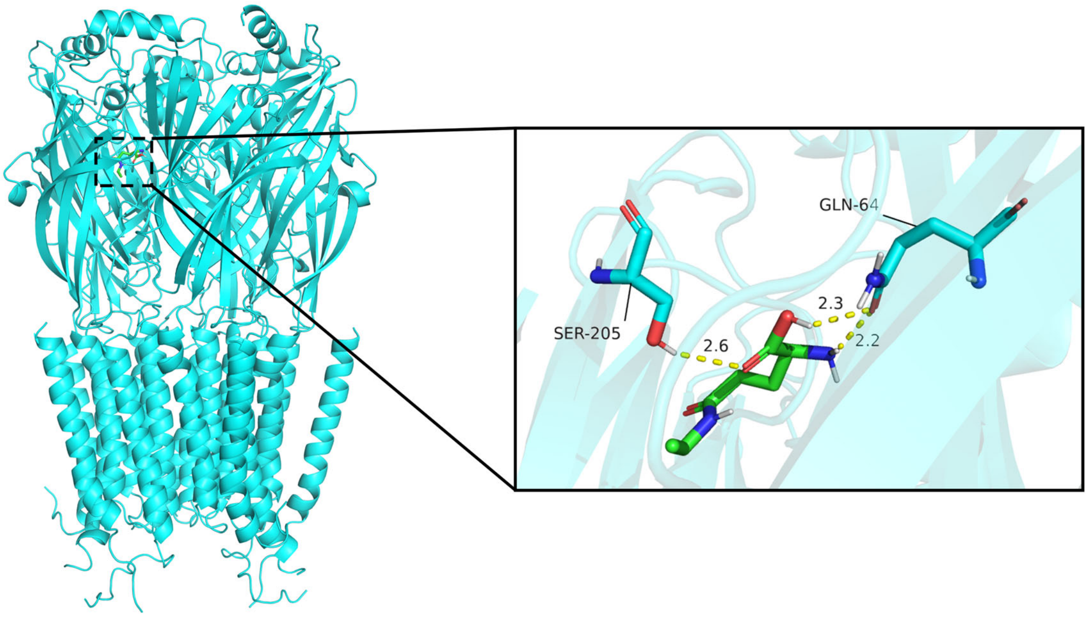

Figure S2. Molecular docking of L-theanine with the human pentameric GABA_A receptor (PDB: 9FG7). The overall receptor structure and enlarged view of the predicted ligand-binding pocket. Yellow dashed lines indicate predicted hydrogen bonds. The predicted binding affinity was −6.2 kcal/mol, with distances labeled in Å. The ligand is shown in green (carbon atoms), red (oxygen atoms), and dark blue (nitrogen atoms). The protein residues are shown in cyan (carbon atoms), with oxygen and nitrogen atoms colored as described above. The labels indicate the specific amino acid residues involved in the binding site.

Table S1. Results of maximum tolerated concentration (MTC) exploration for THEA (n = 30).

Group	Concentration (μg/mL)	Number of deaths (individuals)	Mortality (%)	Phenotype
Control	/	0	0	Normal
Model	/	0	0	Normal
	125	0	0	Similar to the model group
	250	0	0	Similar to the model group
THEA	500	0	0	Similar to the model group
	1000	0	0	Similar to the model group
	2000	0	0	Similar to the model group

Table S2. Concentrations of treatment groups.

Group	Treatment (μg/mL)	PTZ (mM)
Control	-	0
Model	-	5
THEA	L-theanine (500)	5
GABA	GABA (200)	5
MT	Melatonin (125)	5
CAF	Caffeine (50)	5
THEA+GABA	L-theanine (500)<br>GABA (200)	5
THEA+MT	L-theanine (500)<br>Melatonin (125)	5
THEA+CAF	L-theanine (500)<br>Caffeine (50)	5
GABA+CAF	GABA (200)<br>Caffeine (50)	5
MT+CAF	Melatonin (125)<br>Caffeine (50)	5

Table S3. Primer sequences for *gabra1* and *mtnr1aa* in zebrafish used for qRT-PCR.

Primer	sequence (5' to 3')
***β-actin*-F**	TCGAGCAGGAGATGGGAACC
***β-actin*-R**	CTCGTGGATACCGCAAGATTC
***gabra1*-F**	GACGAGTATCGGGCCAGTTT
***gabra1*-R**	TGAGGTTGTTGAGACGGAGC
***mtnr1aa*-F**	TATTTCACCGGGGCTGGAAC
***mtnr1aa*-R**	CATACTGCAAGGAGCCCACA

Table S4. Molecular docking binding affinities of GABA, MT and THEA with GABRA1 and MTNR1AA.

Receptor	Ligand	Affinity (kcal/mol)
GABRA1	GABA	-3.9
	THEA	-5.5
MTNR1AA	MT	-7.8
	THEA	-5.7
